# Supplementary material for: Factor XIIIA—expressing inflammatory monocytes promote lung squamous cancer through fibrin cross-linking
Source: Nat Commun. 2018 May 18;9:1988. doi: 10.1038/s41467-018-04355-w (PMC5959879; doi:10.1038/s41467-018-04355-w)
Supplement: Supplementary file 1 — Supplementary Information [file 41467_2018_4355_MOESM1_ESM.pdf]

Factor XIIIA-Expressing Inflammatory Monocytes Promote Lung Squamous Cancer through Fibrin Cross-Linking

Alessandro Porrello, Patrick L. Leslie, Emily B. Harrison, Balachandra K. Gorentla, Sravya Kattula, Subrata K. Ghosh, Salma H. Azam, Alisha Holtzhausen, Yvonne L. Chao, Michele C. Hayward, Trent A. Waugh, Sanggyu Bae, Virginia Godfrey, Scott H. Randell, Cecilia Oderup, Liza Makowski, Jared Weiss, Matthew D. Wilkerson, D. Neil Hayes, H. Shelton Earp, Albert S. Baldwin, Alisa S. Wolberg, Chad V. Pecot

Supplementary Figure 1

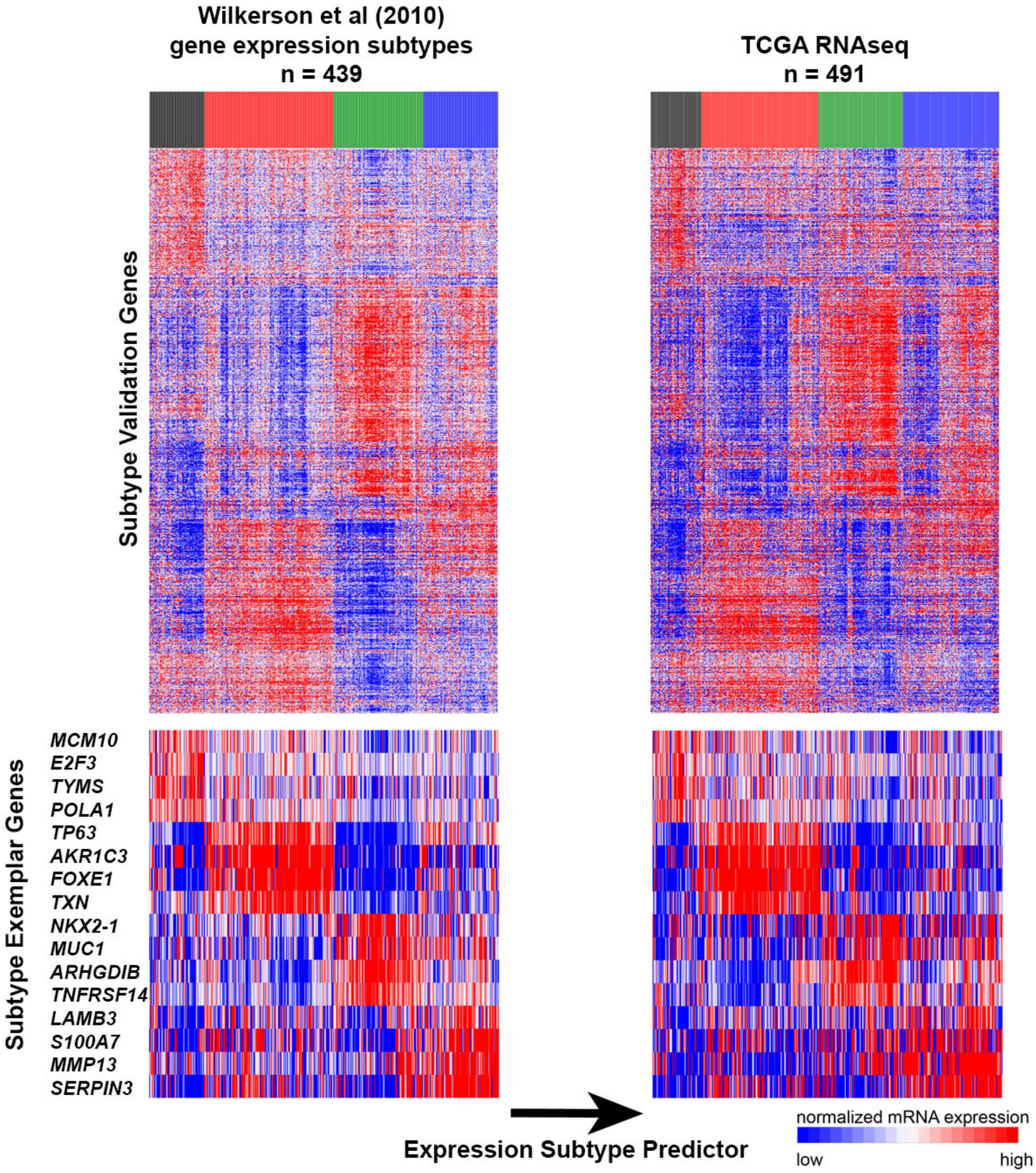

Supplementary Figure 1. Cross-platform comparison between the lung squamous expression subtype classifier applied to the samples of Wilkerson et al and of the TCGA LUSC RNA-seq cohort. The four heat maps correspond to: 1) the genes used by Wilkerson et al for the validation of the four LUSC subtypes using microarray data (top left), 2) the genes used for the validation of the four subtypes for the TCGA LUSC cohort of samples (top right), 3) the subtype exemplar genes used by Wilkerson et al. (2010) (bottom left), and 4) the subtype exemplar genes of the TCGA LUSC samples (bottom right). The four subtypes are identified by colors that correspond with those previously used in the literature as well as in Figure 1. This figure shows the high subtype-specific concordance between exemplar and validation genes, across distinct platforms and cohorts (n = 439 and 491, respectively).

Supplementary Figure 2

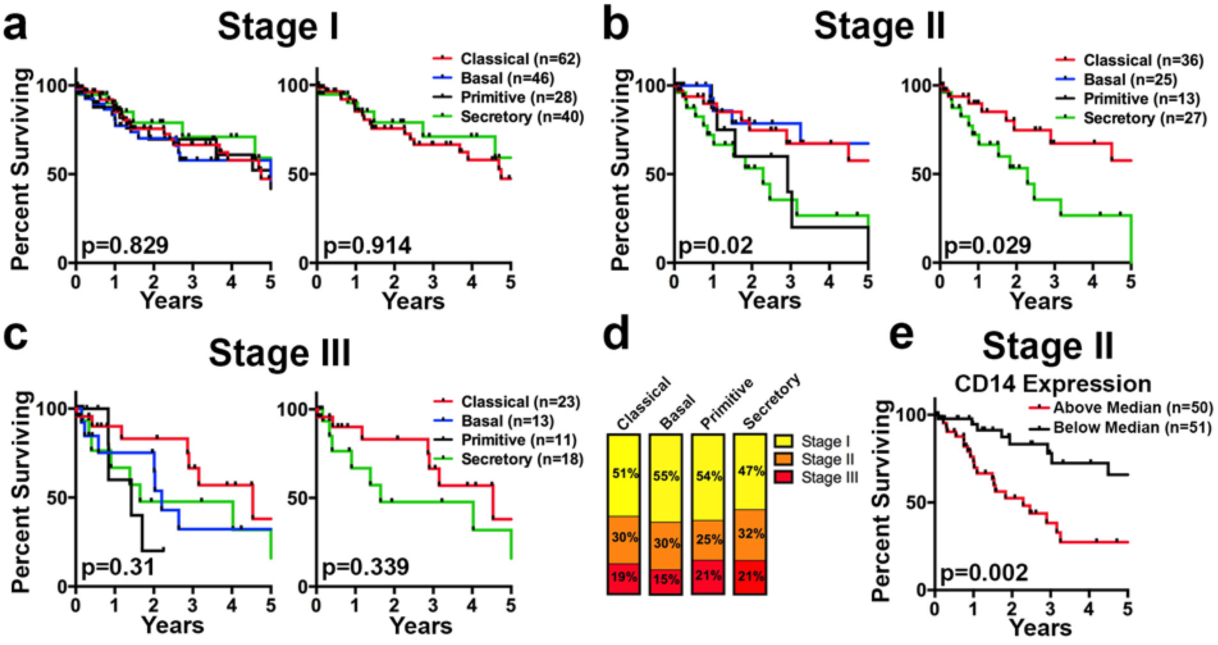

Supplementary Figure 2. Survival data of lung squamous cancer patients from TCGA by clinical stage and CD14 expression. **a**, Kaplan-Meier plots of overall survival for lung squamous carcinoma patients with stage I, **b**, stage II, and **c**, stage III disease according to mRNA subtype (Classical, Basal, Primitive or Secretory). P-values were obtained by using the log-rank test. **d**, Proportion of patients by stage for each mRNA subtype. **e**, Overall survival of lung squamous patients with Stage II disease based on median expression levels of CD14. P-values were obtained by using the log-rank test.

Supplementary Figure 3

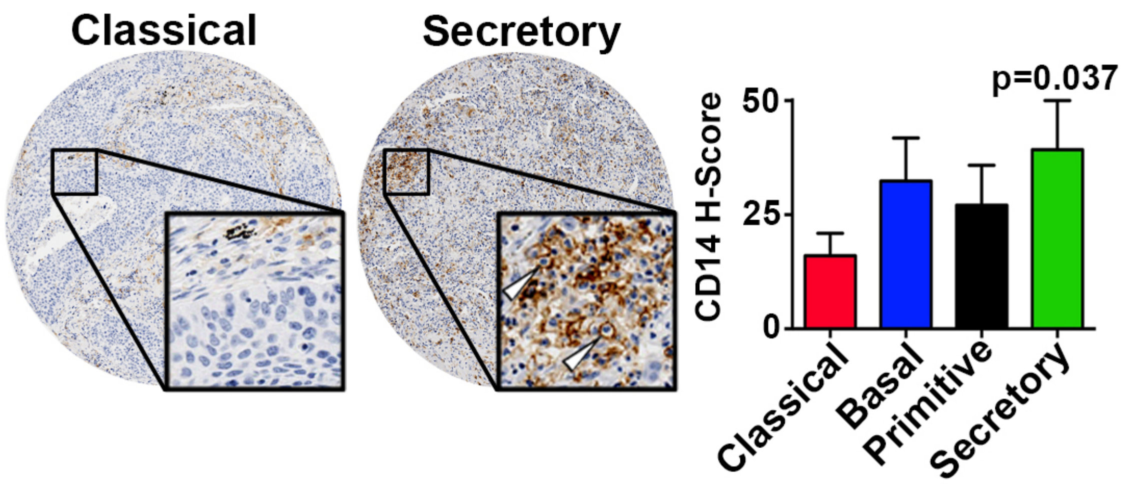

Supplementary Figure 3. Immunohistochemistry for CD14 in a tissue microarray of LUSC subtypes. Representative images of CD14 immunohistochemistry staining (left) of a Classical and Secretory LUSC tumor from the tissue microarray. H-Scores for CD14 (right) for Classical (n=16), Basal (n=11), Primitive (n=6) and Secretory (n=10) tumors. The FDR of the three comparisons vs. Classical are, from left to right: 0.1598, 0.2654 and 0.1110. Data are averages ± s.e.m. P-value was obtained with Student's t-test in comparison to the Classical subtype.

Supplementary Figure 4

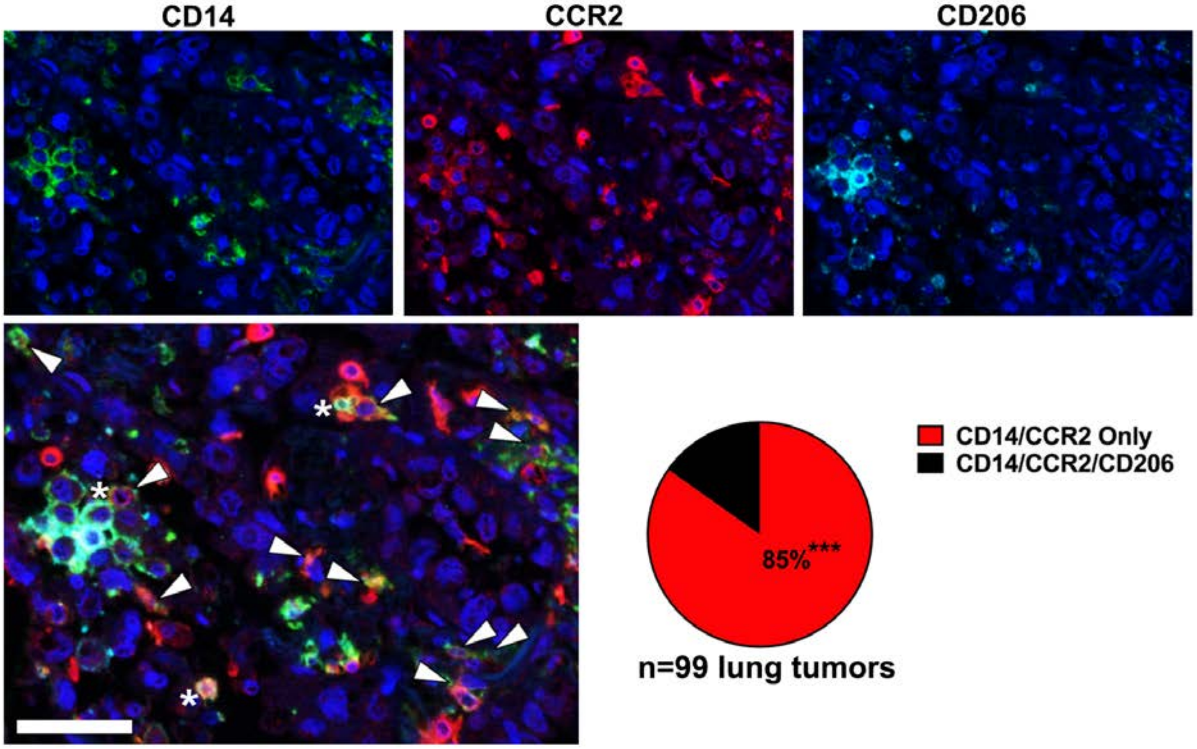

Supplementary Figure 4. Multiplex immunohistochemistry of inflammatory monocytes and CD206+ tumor-associated macrophages. A representative multiplex immunohistochemistry image for CD14 (green), CCR2 (red) and CD206 (light blue) in a lung tumor sample. Dual positive cells are indicated by white arrows, while triple positive cells are next to an asterisk. The pie chart shows the percentage of CD14+/CCR2+ only (n=7,488) and triple-positive CD14+/CCR2+/CD206+ (n=1,342) cells derived from staining a total of 99 lung tumors. Scale bar 50 μm. The statistical significance (of the unbalance between these two cell populations) was calculated using a two-tailed binomial test. *** $P<0.0001$

Supplementary Figure 5

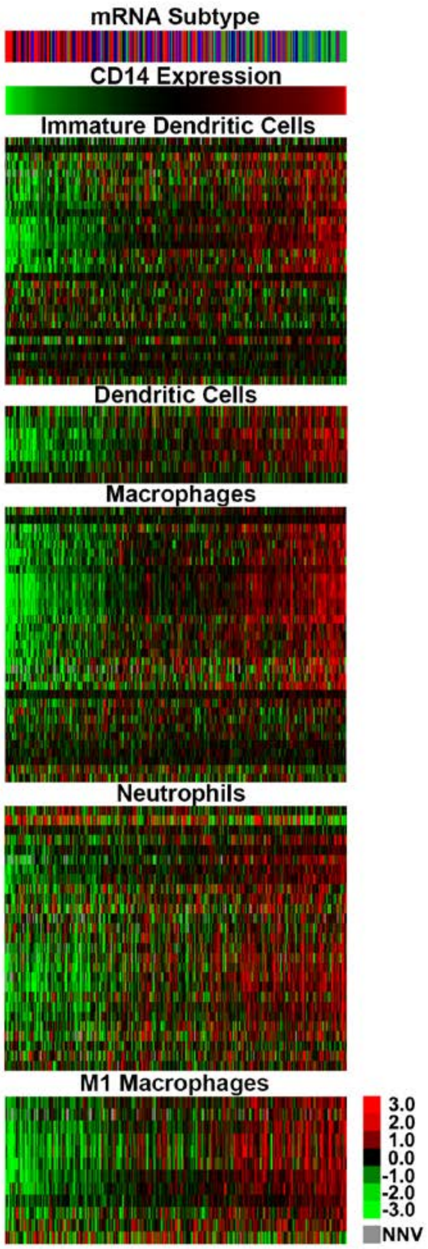

Supplementary Figure 5. CD14+ immune subsets with non-significant p-values for overall survival (OS). Heat maps (with log2 transformed and median centered genes and with samples ordered according to increasing levels of CD14) of the CD14+ populations not shown in Figure 3. These 5 immune cell types have log-rank p-values > 0.05. Note for the heat map figure legend: gray represents 'null normalized values' (NNV).

Supplementary Figure 6

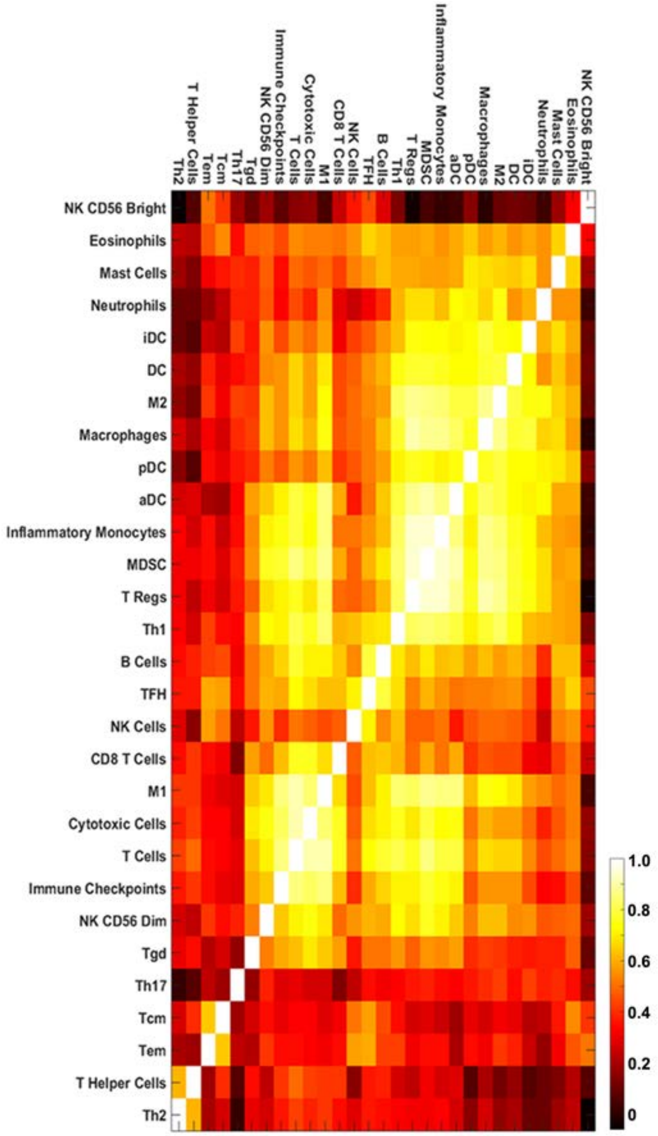

Supplementary Figure 6. Correlations amongst the density scores of the 29 immune cell types analyzed in LUSC. Cross-correlations among immune cell types. Values close to 1 indicate immune cell types that correlate strongly in LUSC patients.

Supplementary Figure 7

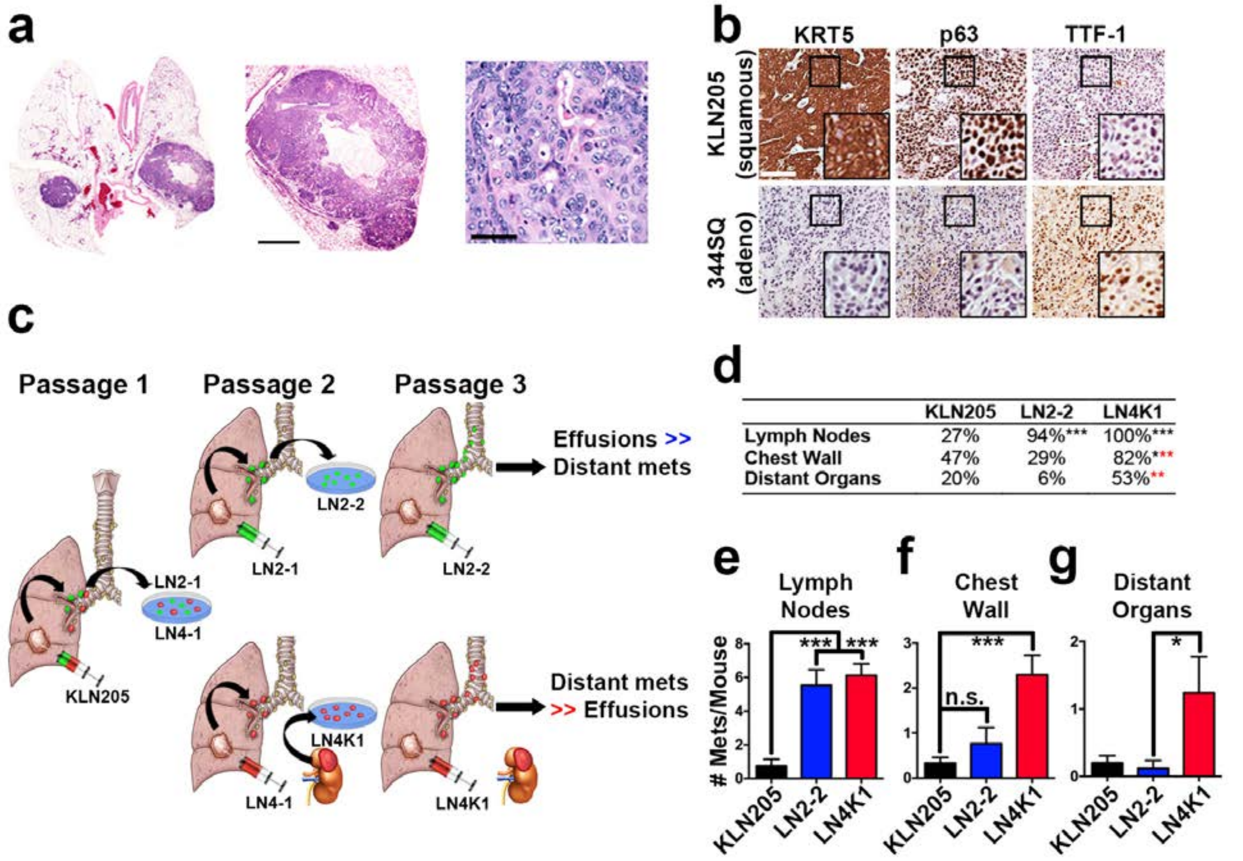

Supplementary Figure 7. Development and characterization of a novel, metastatic, immune-competent LUSC model. a, H&E coronal sections of an orthotopic KLN205 tumor in the left lung as well as a contralateral metastasis in the right lung (left). The orthotopic tumor displays evidence of central necrosis (middle) and poor differentiation (right). Scale bars (middle and right), 1000 and 50 μm, respectively. **b**, Immunohistochemistry stains for KRT5, p63 and TTF-1 comparing KLN205 with a murine lung adenocarcinoma cell line (344SQ). White scale bar: 50 μm. **c**, Schematic of an *in vivo* selection strategy to enrich for sub-clones with unique metastatic properties. **d**, Frequencies of metastasis to thoracic lymph nodes, chest wall and distant organs (e.g. right lung, heart, kidney, liver, diaphragm). P-values were determined using the chi-squared test. Black asterisk: comparison with KLN205; red asterisk: comparison with LN2-2 (tested separately). Relative number of **e**, lymph node, **f**, chest wall, and **g**, distant metastases per mouse compared with the KLN205 parental cell line. Data are averages ± s.e.m. P-values were obtained with Student's t-test. * $P \leq 0.05$, ** $P<0.01$, *** $P<0.0001$

Supplementary Figure 8

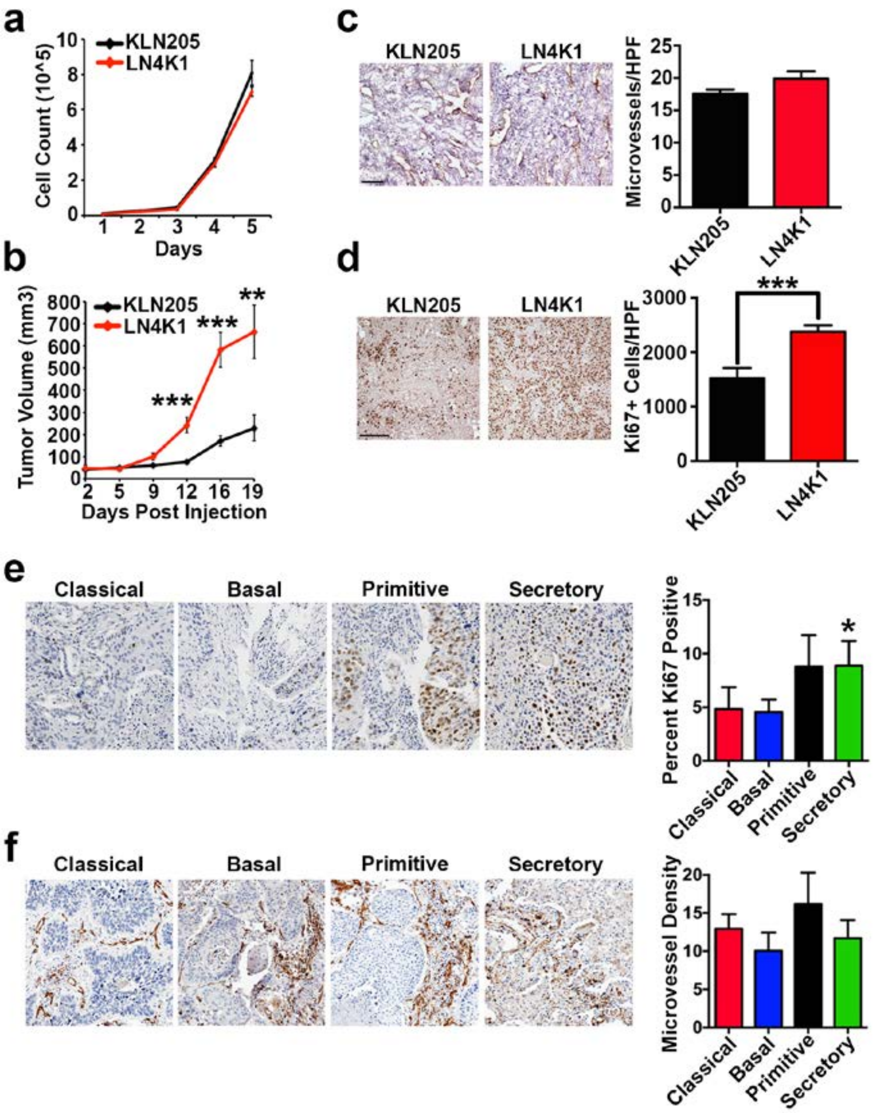

Supplementary Figure 8. Biological indices within the tumor microenvironment of our LUSC mouse model and TCGA LUSC mRNA subtypes. a, Proliferation rates of KLN205 and LN4K1 *in vitro*, and **b**, following subcutaneous injection *in vivo*. Data are averages ± s.e.m. P-values were obtained with Student's t-test, n=5 mice/group. **c**, Representative images of CD31 staining (left) of KLN205 and LN4K1 tumors. Quantitation of the number of microvessels per high power field (right), n=5 tumors/group. Scale bar: 100 μm. **d**, Representative images of Ki67 immunohistochemistry staining (left) of KLN205 and LN4K1 tumors. Scale bar: 50 μm. Quantitation of Ki67 proliferative cells per high power field (right), n=5 tumors/group. Data are averages ± s.e.m. P-values were obtained with Student's t-test. **e**, Representative images of Ki67 immunohistochemistry staining (left) of lung squamous tumors from a tissue microarray according to subtype [Classical (n=14), Basal (n=9), Primitive (n=6) and Secretory (n=12)]. Percent positive Ki67 nuclei (right) are shown according to the molecular subtype. **f**, Representative images of CD31 staining (left) of lung squamous tumors according to the molecular subtype. Quantitation of the number of microvessels per high power field (right). Data of **e**, and **f**, are averages ± s.e.m. P-values of **e** and **f** were obtained using the Mann-Whitney t-test and are relative to the Classical subtype. * $P<0.05$, *** $P<0.001$

Supplementary Figure 9

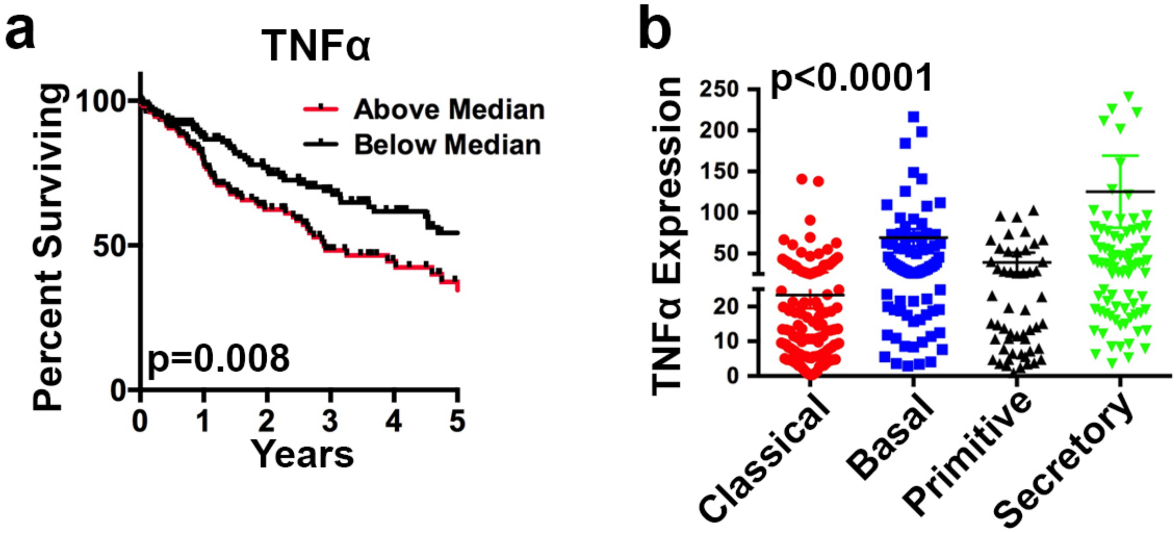

Supplementary Figure 9. Survival analysis and expression levels of TNFα in LUSC. a, Kaplan-Meier plot of OS in LUSC patients segregated by median TNFα expression. The P-value was obtained with a log-rank test. **b**, Dynamic range of TNFα mRNA expression for each LUSC mRNA subtype. The P-value was obtained with one-way analysis of variance (ANOVA).

Supplementary Figure 10

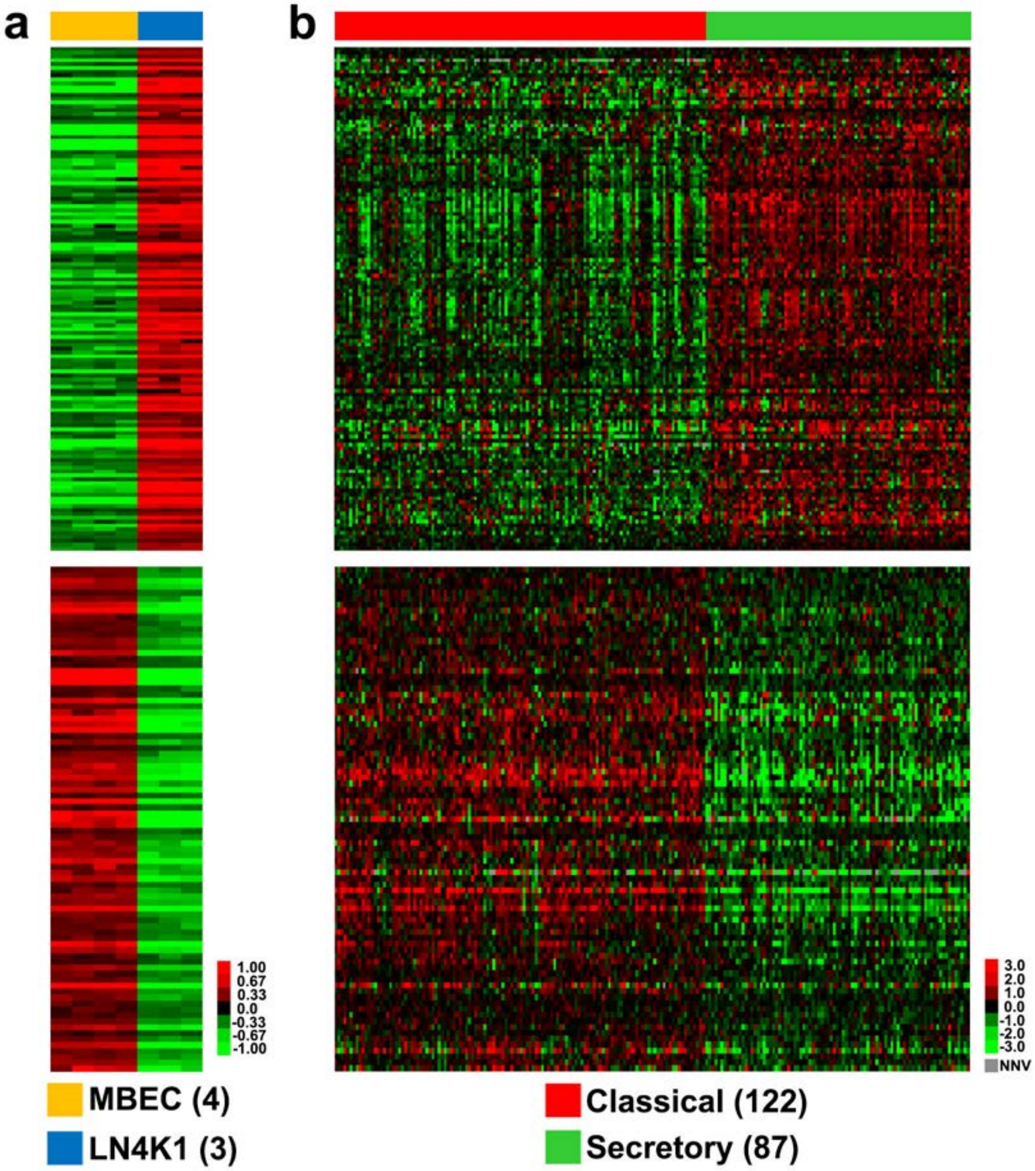

Supplementary Figure 10. Comparison between the gene expression of the murine lung squamous model and TCGA LUSC samples of the Classical and Secretory subtypes. a, Two heat maps showing, after gene-by-gene matching with the TCGA LUSC data set, the comparison between differentially expressed MBEC and LN4K1 samples when the ratio between the medians (= median(MBEC)/median(LN4K1)) of these two groups is below (top) and above 1 (bottom). **b**, Two heat maps of matched genes of the TCGA LUSC data set (subtypes Classical and Secretory). These heat maps are shown according to having their median ratios (= median(Classical)/median(Secretory) below (top) and above 1 (bottom). Data were clustered with respect to the genes of this TCGA LUSC data set (209 samples). For the gene matching and selection criteria used for these two panels, see the Supplementary Methods. Note for the heat map figure legend: gray represents ‘null normalized values’ (NNV).

Supplementary Figure 11

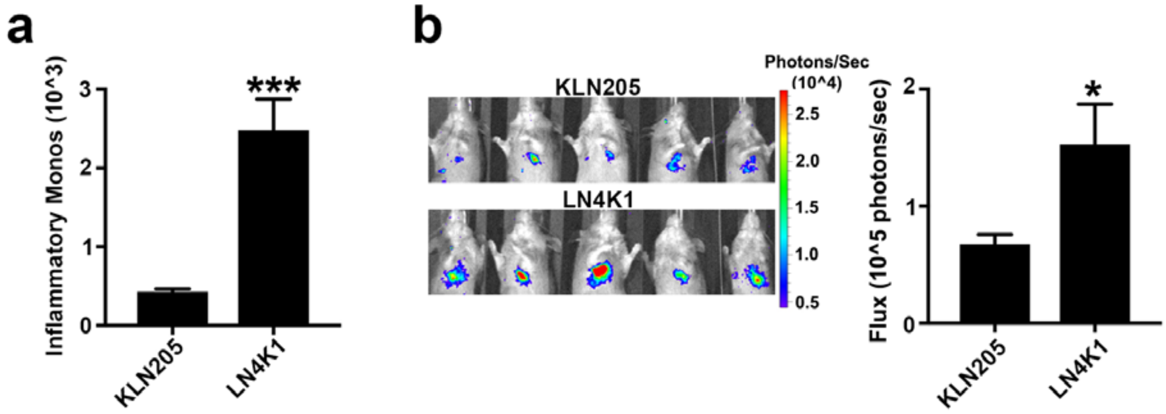

Supplementary Figure 11. LN4K1 recruits significantly more IMs and more rapidly develops metastases. a, Number of IMs per lung lobe between the parental KLN205 and LN4K1 sub-clone, n=12 lobes/group. **b**, Representative images (left) and quantification of luciferase signal (right) obtained 10 days after injection of KLN205 or LN4K1 cell lines expressing luciferase. Data are averages ± s.e.m. P-values were obtained with Student's t-test, n=10 mice/group. * P<0.05, *** P<0.001

Supplementary Figure 12

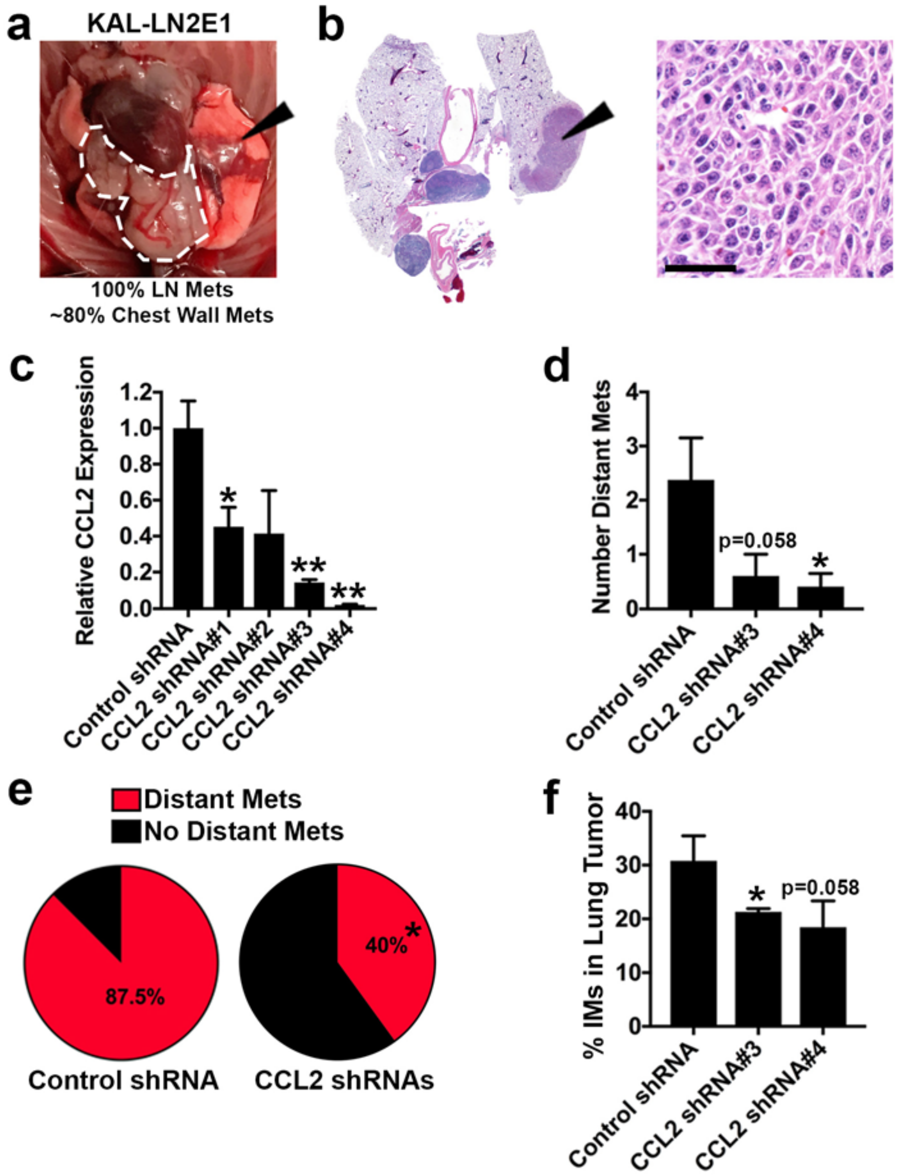

Supplementary Figure 12. CCL2-mediated recruitment of inflammatory monocytes is critical for metastasis in multiple lung squamous mouse models. a, Gross appearance and incidence of lymph node (LN) and distant chest wall metastasis in FVB mice orthotopically injected with KAL-LN2E1 cells. Black arrowhead indicates the primary tumor, dotted white lines encircle LN metastases. **b**, Representative H&E coronal sections of KAL-LN2E1 orthotopic tumors (indicated by the arrowhead) present in the lung. Scale bar=50 μm. **c**, CCL2 mRNA expression levels in KAL-LN2E1 cells stably transduced with shRNA constructs targeting CCL2. **d**, Quantification of the average number of distant metastases per mouse after orthotopic injection of stable shCCL2 KAL-LN2E1 cells, n=5-8 mice/group. **e**, Quantification of the percentage of mice with or without distant metastases by group. P-value determined using Chi-squared. **f**, Quantification of IMs (CD11b+Ly6G-Ly6C^{High}) per lung tumor by flow cytometry (n=4-5 tumors/group). Data are averages ± s.e.m. P-values were obtained with Student's t-test, n.s. = non-significant, * $P \leq 0.05$, ** $P < 0.01$

Supplementary Figure 13

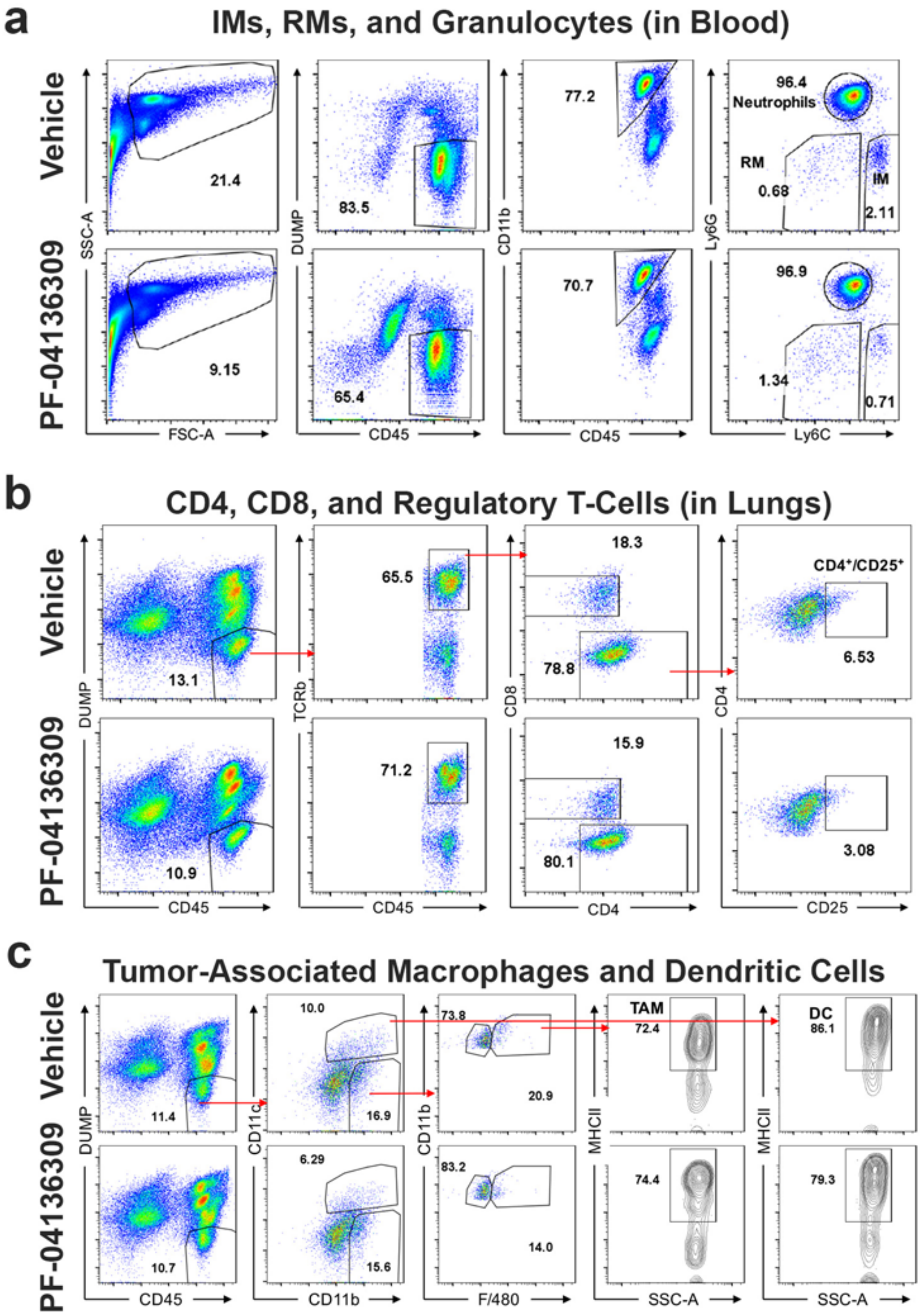

Supplementary Figure 13. Gating strategy and immunologic effects of PF-04136309. a, Representative FACS plots and gating strategy for the detection of inflammatory monocytes (CD11b+Ly6G-Ly6C^{High}), residential monocytes (CD11b+Ly6G-Ly6C^{Low}) and granulocytes (CD11b+Ly6G+Ly6C-) in the blood of LN4K1-bearing mice, **b**, CD4 (TCRb+CD4+CD25-), CD8 (TCRb+CD8+CD25-) and Tregs (TCRb+CD4+CD25+), and **c**, tumor-associated macrophages (SiglecF-CD11b+F480+MHCII+) and dendritic cells (SiglecF-CD11c+F480-MHCII+) in the lung tumors of LN4K1-bearing mice.

Supplementary Figure 14

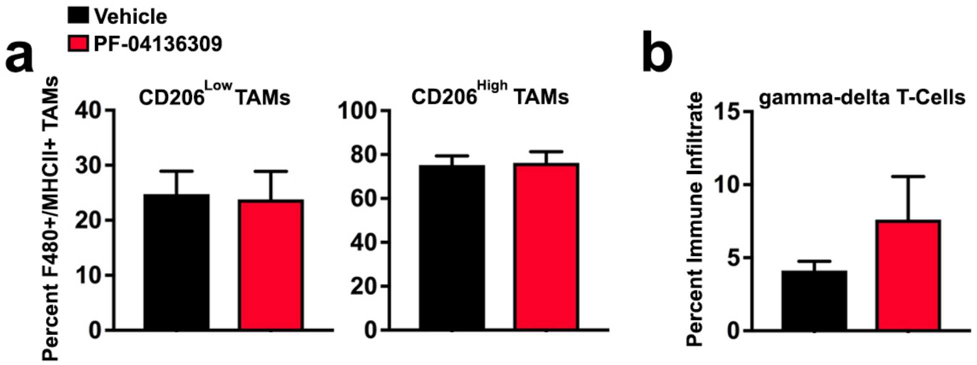

Supplementary Figure 14. Assessment of PF-04136309 effects on tumor-associated macrophage subsets and gamma-delta T-cells. **a**, FACS analysis of percent immune infiltrates for $CD206^{Low}$ and $CD206^{High}$ TAMs (gated on F480+ and MHCII+). **b**, FACS analysis of gamma-delta T-cells (gated on CD45).

Supplementary Figure 15

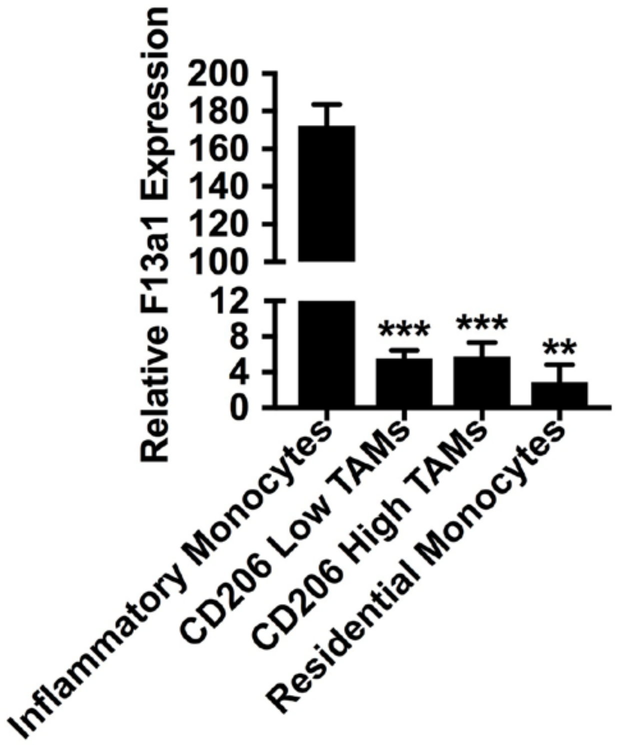

Supplementary Figure 15. Inflammatory monocytes express higher levels of Factor XIIIA than M1 or M2 macrophages. Relative mRNA expression levels of *F13a1* from sorted IMs and RMs (n=2 mice), and TAMs [CD206^{high} (M2) and CD206^{low} (M1)], n=3 mice. Results show the average expression levels for each population. ** *P*<0.01, ** *P*<0.001

Supplementary Figure 16

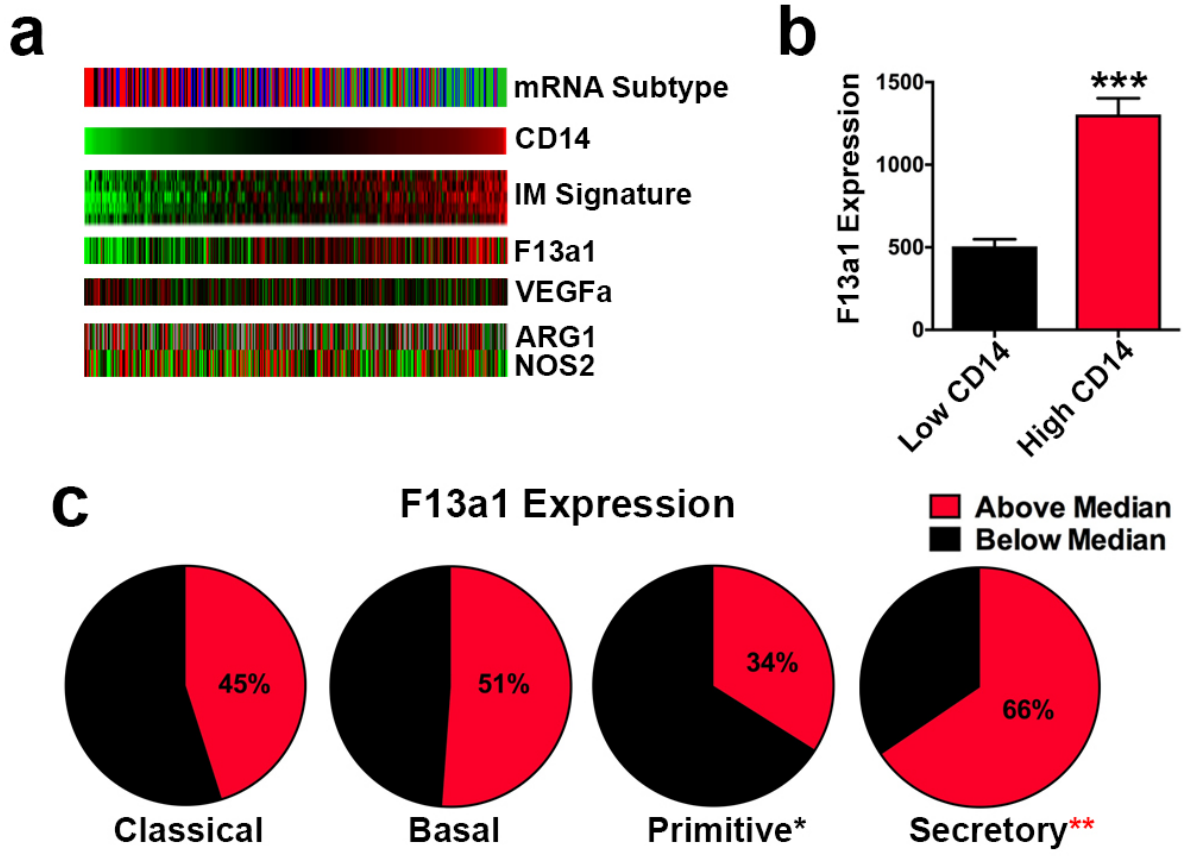

Supplementary Figure 16. F13a1 is highly correlated with the inflammatory monocyte subset. a, Heat maps of the IM signature, F13a1, VEGFa, ARG1 and NOS2 from the TCGA dataset. The sample mRNA subtypes and the (increasing) levels of CD14 are shown on the top. Gene expression levels are log2 transformed and median centered. The full set of markers of IMs is made by this IM signature + CD14. **b**, Bar graph of F13a1 expression levels in the sample groups that express high ('IM enriched' subset, above CD14 median) and low (below CD14 median) levels of CD14. The statistical significance was assessed using a Student's t-test. **c**, Proportion of patients by mRNA subtype that have F13a1 expression levels above (red) or below (black) the median F13a1 expression level. Classical has a binomial test p-value of 0.3193 and an FDR of 0.4257; Basal has a binomial test p-value as well as FDR of 0.9142; Primitive has a binomial test p-value of 0.02701 and an FDR of 0.0540; Secretory has a binomial test p-value of 0.005014 and an FDR of 0.0201. (Black asterisks: significant enrichment below the median, red asterisks: Significant enrichment above the median). * $P < 0.05$, ** $P < 0.01$, *** $P < 0.0001$

Supplementary Figure 17

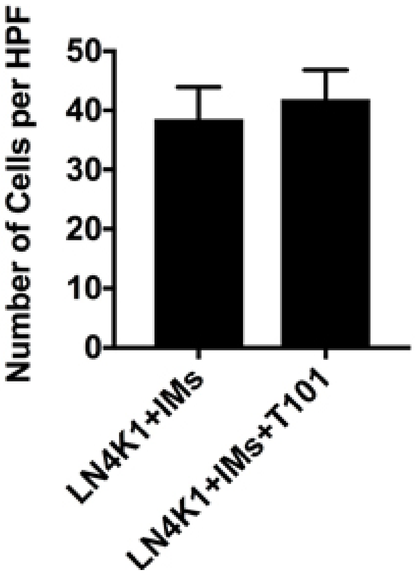

Supplementary Figure 17. Invasive properties of lung squamous cell lines in a Factor XIIIA-independent context. Invaded LN4K1 cells per high power field (HPF) 24 hours after seeding into Matrigel co-cultured with IMs with or without T101 (50 μM).

Supplementary Figure 18

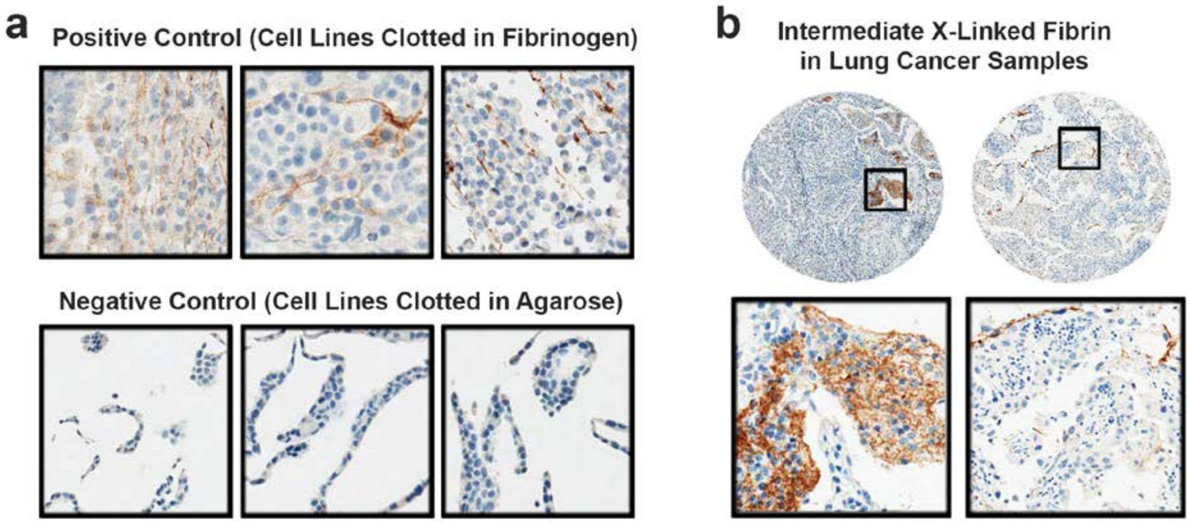

Supplementary Figure 18. Development and validation of an immunohistochemistry approach to detect cross-linked fibrin. a, Validation in cancer cell lines clotted in fibrinogen/thrombin (positive control, top) of a novel monoclonal antibody (Zedira, A076, 1:1,500 dilution) that binds to a cross-linked fibrin epitope. Cell lines clotted in agarose (bottom) were used as a negative control. **b**, Lung squamous tumors showing intermediate staining patterns for cross-linked fibrin.
