# Supplementary material for: Factor XIIIA—expressing inflammatory monocytes promote lung squamous cancer through fibrin cross-linking
Source: Nat Commun. 2018 May 18;9:1988. doi: 10.1038/s41467-018-04355-w (PMC5959879; doi:10.1038/s41467-018-04355-w)
Supplement: Supplementary file 3 — Description of Additional Supplementary Files [file 41467_2018_4355_MOESM3_ESM.pdf]

Description of Additional Supplementary Files

File Name: Supplementary Data 1
Description: Clinical data elements for lung squamous carcinoma patients from the Cancer Genome Atlas.

File Name: Supplementary Data 2
Description: All 403 genes with statistically significant hazard ratios for overall survival from the Secretory subtype.

File Name: Supplementary Data 3
Description: All genes from the ingenuity pathway analysis that attributed to the leukocyte migration pathway.

File Name: Supplementary Data 4
Description: Gene ontology analyses with significant p-values for the lung squamous carcinoma subtypes shown.

File Name: Supplementary Data 5
Description: Gene set enrichment analyses for the top signatures amongst the Secretory subtype of lung squamous carcinoma

File Name: Supplementary Data 6
Description: Clinical data elements for the patients with tumors on the tissue microarray that have subtype calls.

File Name: Supplementary Data 7
Description: Table showing gene name, EntrezGeneID, and cell types used for the immunogenomic analyses.

File Name: Supplementary Data 8
Description: Differentially expressed genes with Affymetrix microarray profiling with fold changes between mouse bronchial epithelial cells, KLN205 and LN4K1 cell lines.

File Name: Supplementary Data 9
Description: Significant upstream regulators of the Affymetrix microarray when using ingenuity pathway analysis.

File Name: Supplementary Data 10
Description: Genes commonly increased and decreased in the LN4K1 sub-clone and Secretory subtype of lung squamous carcinoma.

File Name: Supplementary Data 11
Description: Table containing the mRNA subtype assignments for each of the lung squamous samples in the Cancer Genome Atlas.

File Name: Supplementary Data 12
Description: The list of probes used for the Affymetrix microarray analysis, together with the available Affymetrix annotation.

File Name: Supplementary Movie 1
Description: A three-dimensional visualization of an inflammatory monocyte stained for FXIIIA (red) and DAPI (blue).
